# Does postural stability differ between adolescents with idiopathic scoliosis and typically developed? A systematic literature review and meta-analysis

**DOI:** 10.1186/s13013-018-0163-1

**Published:** 2018-09-03

**Authors:** Marlene Dufvenberg, Fisayo Adeyemi, Isabelle Rajendran, Birgitta Öberg, Allan Abbott

**Affiliations:** 1 Department of Medical and Health Sciences, Division of Physiotherapy, Linkoping University, 581 83 Linkoping, Sweden; 2 Department of Clinical and Rehabilitation Services, Faculty of Health Science and Medicine, Bond Institute of Health and Sport, Bond University, 2 Promethean Way, Robina, QLD 4226 Australia

**Keywords:** Adolescent idiopathic scoliosis, Postural balance, Postural control, Postural stability, Posturography, Force plate, Centre of pressure, Sway area, Anteroposterior, Mediolateral

## Abstract

**Background:**

Postural stability deficits have been proposed to influence the onset and progression of adolescent idiopathic scoliosis (AIS). This study aimed to systematically identify, critically evaluate and meta-analyse studies assessing postural stability during unperturbed stance with posturography in AIS compared to typically developed adolescents.

**Methods:**

Studies from four electronic databases (PubMed, Scopus, CINAHL, PEDro) were searched and case-control methodological quality assessed using a risk-of-bias assessment tool and a posturography methodological quality checklist. Pooled data regarding centre of pressure (COP) parameters such as sway area, Mediolateral (ML) and Anteroposterior (AP) position and range were compared for AIS and typically developed adolescents using Cohen’s *d* effect size (ES) and homogeneity estimates.

**Results:**

Eighteen studies for quality analysis and 9 of these for meta-analysis were identified from 971 records. Risk-of-bias assessment identified 6 high, 10 moderate and 2 low risk-of-bias studies. The posturography methodological quality checklist identified 4 low, 7 moderate and 7 high-quality studies. Meta-analysis was performed for sway area whereas ML and AP are presented in three different meta-analyses due to divergent measurement units used in the studies: ML position 1 (MLP1), ML position 2 (MLP2) and ML range (MLR); AP position 1 (APP1), AP position 2 (APP2) and AP range (APR). Cohen’s *d* showed a medium ES difference in sway area *0*.*65*, 95% CI (0.49–0.63), whereas ML showed no (MLP1, MLP2) and large (MLR) ES differences; MLP1 *0.15*, 95% CI (0.08–0.22); MLP2 *0.14*, 95% CI (0.08–0.19); and MLR 0.94, 95% CI (0.83–1.04). Cohen’s *d* for AP showed small ES (APP1) and large ES difference (APP2 and APR); APP1 *0.43*, 95% CI (0.31–0.54); APP2 *0.85*, 95% CI (0.72–0.97); and APR *0.98*, 95% CI (0.87–1.09). Cochran’s Q and Higgins *I*^2^ showed homogeneity between studies.

**Conclusions:**

There is moderate quality evidence for decreased postural stability in AIS measured as COP parameters sway area, ML and AP range with a positional shift posteriorly in the sagittal plane. The findings support studying postural stability in early stage AIS and also prospectively identify cause and effect of the curvature as well as effectiveness of postural control interventions in the prevention of scoliosis progression.

## Background

Postural control involves the body’s position in space for dual purposes of stability and orientation [1]. Postural stability is often described as the ability to control the centre of body mass (COM) relative to the base of support (BOS) [1] while postural orientation is the ability to maintain an appropriate relationship between the body segments and between the body and the environment for a task [2–4]. COM is often used interchangeably with the term centre of gravity (COG) [5]. Postural control in fairly predictable and non-challenging conditions, e.g. standing quietly is defined as steady-state balance [1]. Postural sway always occurs during quiet standing because of our vertical posture which is inherently unstable due to our relatively high COM [3, 6]. Postural control is a complex skill based on the interaction of multiple dynamic sensorimotor and cognitive systems to maintain postural stability under static and dynamic conditions [1, 2, 7]. Pathology to any of the underlying systems will result in different, context-specific postural deficits [7].

All neuromuscular disorders that act on the growing body, particularly during the rapid pubertal growth period, can lead to the development of scoliosis [8]. Adolescent idiopathic scoliosis (AIS) is a three-dimensional deformity of the spine and trunk with a lateral deviation of ≥ 10° which can occur between the ages of 10 and 17. It is the most common cause of spinal deformity in otherwise healthy adolescents [9–11]. AIS affects 0.47 to 5.2% of the general population, with an apparent gender dominance, rising with age and severity, with a female to male ratio from 1.5:1 to 10:1 [10–12]. This condition is associated with a higher incidence of back pain and discontent with body image and can in severe cases lead to pulmonary functional deficits [13].

The pathogenesis of AIS remains unknown but is considered to be multifactorial [14–16]. A disharmony between autonomic and somatic nervous systems has been hypothesised to cause changed regulation of somatosensory input and motor output [8, 17]. These changes may cause postural stability deficits affecting the onset and curve progression [18]. Previous studies on changes in postural stability deficits for individuals with AIS compared to typically developed adolescents (CON) have shown inconsistent results from significant differences [19] to no differences in balance tasks [20, 21].

There are a variety of methods to assess postural control during different conditions and ages [22–24]. Posturography, bipedal static task on force plate (force platform), is the most used devise to provide an indirect assessment of changes in postural sway to gain a better understanding of quiet standing balance [22, 24, 25]. Force plate measures ground reaction forces (GRF) that represent the sum of the pressure distributed under the foot. Centre of pressure (COP) refers to the point at which the pressure would be concentrated [5, 26]. Postural stability can thus be quantified using COP parameters derived from GRF to assess alterations in balance [22, 25, 26]. Posturography has been used to determine if postural stability is changed in AIS and if type and location of scoliosis affect progression [27].

Various COP parameters calculated as sway area, mediolateral (ML), and anteroposterior (AP) change of position and range have been derived from COP data [22, 24, 26]. Sway area refers to body oscillations, often described as a 95% ellipse area which is expected to enclose approximately 95% of the points on the COP path [22, 24]. ML and AP assessed as position defines an object’s location [22] whereas range is the maximum distance between any two points on the COP path relative to a baseline value or axis [24]. Mean amplitude of COP is an average value over all data points collected in a trial and is a representative measure of postural control [24, 26].The movement of COP in the positive direction, according to right-hand coordinate system, is towards the right in the frontal plane whereas the AP direction indicates a forward displacement in the sagittal plane from the central body position [28]. Increased values are an indication of decreased postural stability [22, 26]. The reliability of COP measures have been criticised but can be used as a reliable tool for investigating general postural stability under specific conditions [29, 30].

The purpose of this review was to identify, critically evaluate and meta-analyse studies assessing postural stability during unperturbed stance with posturography in AIS compared to CON. We hypothesised that AIS would have decreased postural stability compared to typically developed adolescents measured as COP parameters sway area, ML and AP position and/or range.

## Methods

### Data sources and searches

Studies published until the end of 2016 was retrieved from a search of four electronic databases: PubMed, Scopus, CINAHL and PEDro in April–May 2017 to identify eligible studies. The authors in consultation with an academic librarian designed the search strategy to identify relevant studies comparing postural stability within AIS compared to a control group with typically developed adolescents (CON). The following main search terms were used: (“scoliosis” AND “adolescent” AND “postural AND sway” OR “postural AND stability” OR “postural” AND “control” OR “postural” AND “balance”), appropriate to each database (Table 1). A reference list search was carried out on included full-text studies.

**Table 1:** Overview of search strategy and retrieved studies

Database Date of search dd/mm/yy	Search period included dd/mm/yy	Search terms	Total number	Number from title and abstract	Duplicated	New studies
Scopus 18/04/17	31/12/16	((TITLE-ABS-KEY(idiopathic AND scoliosis) OR (idiopathic AND scolioses) OR AIS OR scoliosis OR (spinal AND deformity) OR (spinal AND deformities) OR scoliotic OR scolioses OR (spinal AND curvature) OR (spinal AND curvatures))) AND (TITLE-ABS-KEY ((postural AND sway) OR (postural AND stability) OR (postural AND function) OR (postural AND control) OR (postural AND behaviour) OR (postural AND behavior) OR (postural AND performance) OR (postural AND regulation) OR (postural AND strategy) OR (postural AND strategies) OR (postural AND dysfunction) OR (postural AND dysfunctionality) OR (body AND balance) OR (body AND sway) OR (postural AND control AND system) OR (postural AND balance) OR (body AND equilibrium)))) AND (TITLE-ABS-KEY ((adolescen* OR youth* OR teenager*)))	458	23	–	0
PubMed 06/04/17	23/09/16	((((((((Scoliosis OR spinal curvatures[MeSH Terms]))) OR ((Idiopathic Scoliosis OR AIS OR Scoliosis OR spinal deformity OR scoliotic OR scolioses OR spinal curvatures)))) AND ((((Adolescent OR Adolescence OR youth OR teenager[MeSH Terms]))) OR ((Adolescent OR Adolescence OR youth OR teenager))))) AND ((((Postural sway OR postural stability OR postural function OR postural control OR postural behaviour OR postural performance OR postural regulation OR postural strategy OR postural dysfunction OR body balance OR body sway OR postural control system OR postural balance OR body equilibrium))) OR postural balance[MeSH Terms]))	396	16	300 249 Scopus, 56 CINAHL 1 PEDro	0
CINAHL 18/05/17	23/09/16	(Idiopathic Scoliosis OR AIS OR Scoliosis OR spinal deformity OR scoliotic OR scolioses OR spinal curvatures) AND (Postural sway OR postural stability OR postural function OR postural control OR postural behaviour OR postural performance OR postural regulation OR postural strategy OR postural dysfunction OR body balance OR body sway OR postural control system OR postural balance OR body equilibrium) AND (Adolescent OR Adolescence OR youth OR teenager)	81	1	107 51 Scopus 56 PubMed 0 PEDro	0
PEDro 18/05/17	31/12/16	Scoliosis and Clinical trial	36	0	1 PubMed	0
All databases			971		357	
After duplicates			614			
Retrieved for full text				23		0

### Screening and selection

The inclusion criteria were as follows: (1) adolescents aged 10–18 years; (2) use of force plate to measure postural stability during stance; (3) inclusion of one or more reported parameters: mean sway area, mean position and/or range in ML and AP direction; (4) AIS and typically developed adolescents; (5) case-control studies; and (6) studies published in English. The exclusion criteria were as follows: (1) non-idiopathic scoliosis; (2) non-healthy subjects (other neuromuscular, neurological, congenital or trauma-related co-morbidities); (3) patients who have undergone scoliosis correctional surgery or in brace; (4) an absence of CON; and (5) measurements during perturbed stance. Further exclusion criteria for meta-analysis were (6) divergent measurement units or discrete values and graphs missing, (7) use of two force plates, (8) procedure with feet or heels together, (9) and high severity scoliosis group, Cobb > 60°.

**Fig. 1:**
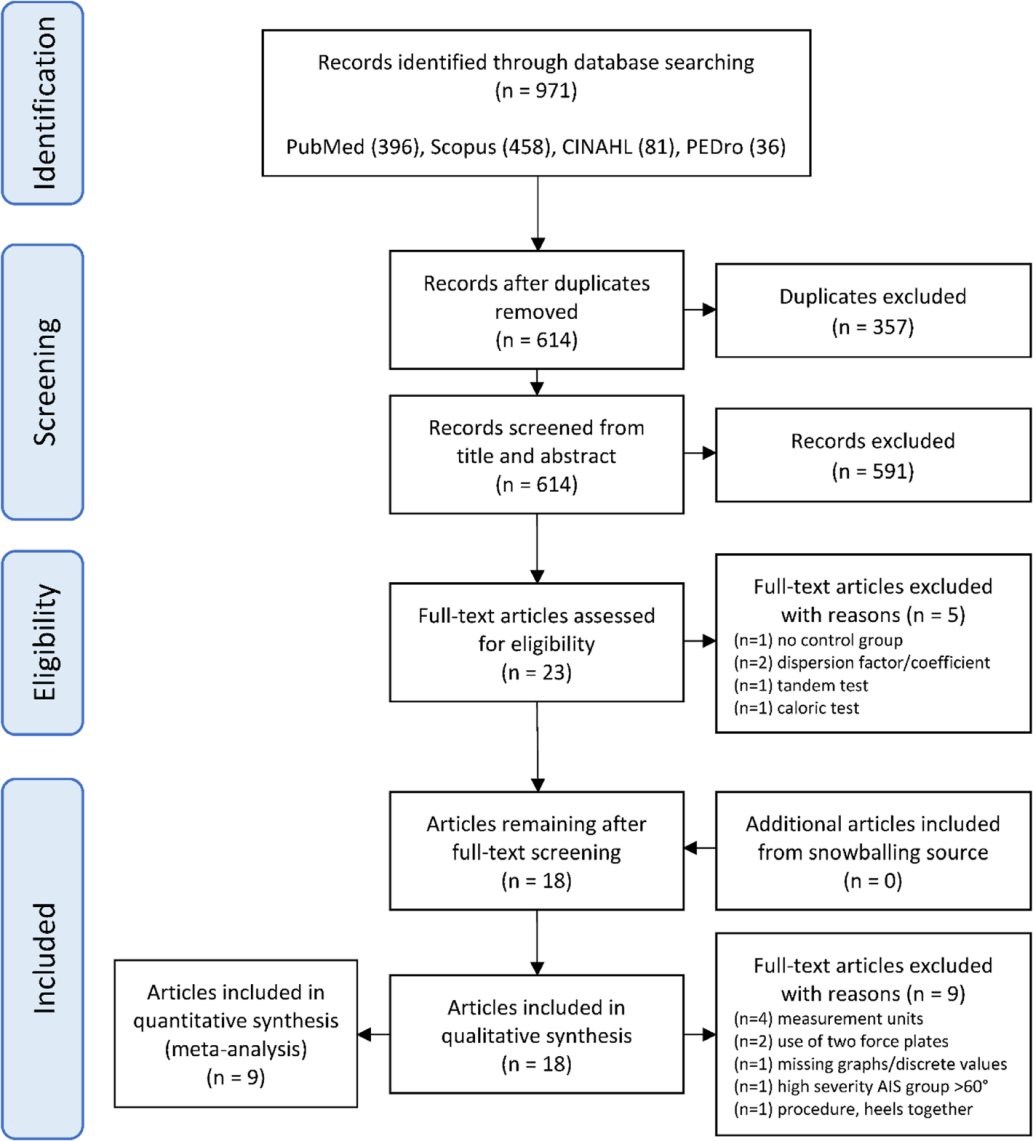
PRISMA flow diagram describing selection process for included studies

The study followed the PRISMA reporting guidelines and a flow diagram was used to document the study screening and selection process [31] (Fig. 1). From the initial studies, identified duplicates were removed. Two assessors (M.D., F.A.) manually screened the titles and abstracts for relevance and likelihood of meeting the inclusion and exclusion criteria. Three appraisers (M.D., F.A., I.R.) evaluated remaining studies using the inclusion and exclusion criteria to yield studies for the qualitative and quantitative synthesis.

### Critical appraisal

Methodological quality was assessed using two critical appraisal tools. A risk-of-bias assessment tool for case-control methodology (Table 2) was chosen due to its population specificity [32]. A six-item scoring system was performed for description and validity of key information to facilitate categorisation of the risk-of-bias impact on the quality of studies, one point for each criterion with minimal score of 0 and a maximal score of 6 possible. Cut off values suggested by Schlösser et al. [32] were not used for exclusion of studies. Included studies were instead ranked according to the following 0–2 as high, 3–4 moderate, and 5–6 being a low risk of bias. The posturography methodological quality checklist (Table 3) was selected to consider internal and external validity and to identify differences that may explain heterogeneity between studies [33]. The total number of items with positive points were divided by the total number of items and multiplied by 100 to produce a quality score for each domain. An overall measure of quality was found by averaging each domain score. Studies were further ranked according to their total score: ≤ 49 = low, ≥ 50 = moderate and ≥ 80 = high quality. Three independent blinded appraisers undertook the assessment (M.D., F.A., I.R.). For any disagreements between appraisers’ discussions, a fourth reviewer (A.A.) participated to reach consensus.

**Table 2:** Risk-of-bias assessment tool for case-control methodology

Item	Scoring
Selection:	
1. Is the control group representative for normal adolescents?	1 = Community control; 0 = Hospital controls; 0 = No description of source
2. Was other pathology excluded that possibly influences the outcome?	1 = Yes; 0 = No or no description
Comparability:	
3. Were the same in- and exclusion criteria (except for the spinal deformity) used for AIS and healthy adolescents?	1 = Yes; 0 = No or no description
Exposure/outcome:	
4. Were the observers blinded to AIS/healthy adolescent status?	1 = Yes; 0 = No or not documented
5. Was the data collection performed in the same standardised way for AIS cases and healthy adolescents?	1 = Yes; 0 = No or not documented
6.^a^ Was the primary outcome parameter for AIS cases and healthy adolescents available?	1 = Available for > 90% of AIS and healthy adolescents; 0 = Available for < 90% of AIS or healthy adolescents

^a^Modified for AIS population

**Table 3:** The posturography methodological quality checklist

Item	Description	Scoring
Internal validity		
1	Indicates reliability of outcome measures	A positive point was assigned if a minimum sampling duration of 90 s and/or 3–5 reps was used
2	Clear presentation of balance assessment	A positive point was assigned if replication of the experiment is possible based on the information in the article
3	Correction for confounding effect on dependent variables	Positive points were assigned if confounders were taken into account, or appropriate matching on these variables was performed
		3a Study controls for age
		3b Study controls for gender
		3c Study controls for height
		3d Study controls for body mass
Statistical validity		
4	The use of appropriate statistical tests	A positive point was assigned if appropriate tests were used to assess differences in balance
5	Adequate sample size	A positive point was assigned if a minimum of 20 subjects per group were included
External validity		
6	Sufficient information about the subject’s characteristics	Positive if information was provided about
		6a Adequate information regarding age
		6b Adequate information regarding gender
		6c Adequate information regarding height
		6d Adequate information regarding body mass
		6e Adequate information regarding severity of curve (Cobb angle)

### Data extraction and synthesis

Key data regarding the study settings, participant demographics, study protocol, COP parameters, results and conclusions were extracted and tabulated. If discrete values were not reported, estimated values were extracted from graphs or figures. A critical qualitative synthesis was conducted with a consideration of risk-of-bias assessment and posturography methodology (Tables 2 and 3).

To evaluate the quantitative differences in postural stability in AIS compared with CON, meta-analysis was conducted. Increased values for one of the groups expressed decreased postural stability. Means and standard deviations (SD) for posturography data were collected from each study; if multiple AIS subgroups were presented, combined means were calculated. All measures were converted to square millimetres and millimetres. Confidence interval (CI) and standard error/of measurements (SE and SEM) were converted to SD (two-tailed, 95% CI). Effect sizes (ES) (Cohen’s *d*) [34] for COP data collected in each study was calculated using an online calculator [35, 36]. The statistical calculations for meta-analysis and ES were performed using Microsoft Office Excel meta-analysis package [37]. The meta-analysis package included a built-in random-effects model considering ES and how homogeneous the data was from the different studies. As a result, the overall (pooled) ES for these studies and a confidence interval (CI) of 95% could be calculated [37]. According to Cohen [34], *d* = 0.20 is considered as a small, *d* = 0.50 medium and *d* = 0.80 a large ES difference*.*

Cochran’s Q and Higgins’s *I*^2^ statistics were calculated to assess homogeneity across studies using the random-effects model [38, 39]. Cochran’s Q was compared to a chi-square critical values table (*χ*^2^), alpha level *p* < 0.05, where degrees of freedom are the number of studies in the analysis minus one. If the Cochran’s Q value is under the chi-square value, we accept the null hypothesis that all studies are homogeneous [38]. Higgin’s *I*^2^ is an estimate of the percentage of total variability that can be attributed to the variation between studies [40]. Thus, percentages of Higgins’s *I*^2^ around 25% (*I*^2^ = 25), 50% (*I*^2^ = 50) and 75% (*I*^2^ = 75) were classified as low, medium and high heterogeneity, respectively. In this review, *I*^2^ below 25% suggests homogeneity [38, 41].

In this review, seven meta-analyses were calculated from COP parameters. One represents sway area while ML and AP are each presented as three different meta-analyses due to divergent measurement units used in the included studies: ML position 1 (MLP1), ML position 2 (MLP2) and ML range (MLR); AP position 1 (APP1), AP position 2 (APP2) and AP range (APR), where APP2 describes the change in position from a location from the back of the heels and leaning forward in opposite to APP1 where the location is estimated from the centre point of support and backwards. The three different categorisations were performed likewise for MLP1 and MLP 2 to minimise the risk of heterogeneity in the meta-analyses.

## Results

### Literature review

Electronic database searches yielded 971 potential studies (Table 1). After title and abstract review and removal of duplicates, 23 studies were retrieved in full text (Fig. 1). The full-text review excluded 5 studies due to the following reasons: no CON, dispersion factor or coefficient as parameter, during caloric, and during tandem test. This resulted in 18 studies for inclusion in the synthesis component of the study from the most recent to the oldest published [42–59]. A search from reference lists yielded no additional studies (Fig. 1).

### Critical appraisal of methodological quality

The scoring of methodological quality for each study using a risk-of-bias assessment tool is outlined in Table 4. After evaluating 18 studies, 6 [44, 48, 50, 54, 56, 57] showed a high risk of bias (total score 2), 10 [43, 45–47, 51–53, 55, 58, 59] showed a moderate risk of bias (total score 3–4) and two [42, 49] showed a low risk of bias (total score 5). Nil studies scored the lowest risk of bias (total score 6) or the highest risk of bias (total score 0) possible. Four out of 18 studies [42, 46, 47, 49] met the criteria of the same inclusion and exclusion criteria for CON and AIS.

**Table 4:** Overview of risk-of-bias assessments of all included studies and total score

Author/year	Risk-of-bias selection		Risk-of-bias comparability	Risk-of-bias exposure/outcome			Total score 0–6 points
	Item 1	Item 2	Item 3	Item 4	Item 5	Item 6	
Sahli et al. 2013 [42]	1	1	1	0	1	1	5
Park et al. 2013 [43]	0	1	0	0	1	1	3
De Santiago et al. 2013 [44]	0	0	0	0	1	1	2
Stylianides et al. 2013 [45]	0	1	0	0	1	1	3
Gruber et al. 2011 [46]	0	1	1	0	1	1	3
Dalleau et al. 2011 [47]	0	1	1	0	1	1	4
Valles et al. 2009 [48]	0	1	0	0	1	0	2
Beaulieu et al. 2009 [49]	1	1	1	0	1	1	5
Zabjek et al. 2008 [50]	0	0	0	0	1	1	2
Dalleau et al. 2007 [51]	0	1	0	0	1	1	3
Simoneau, et al. 2006 [52]	0	1	0	0	1	1	3
Chow et al. 2006 [53]	1	1	0	0	1	1	4
Simoneau et al. 2006 [54]	0	0	0	0	1	1	2
Allard et al. 2004 [55]	1	1	0	0	1	1	4
Silferi et al. 2004 [56]	0	0	0	0	1	1	2
Nault et al. 2002 [57]	0	0	0	0	1	1	2
Chen et al. 1998 [58]	0	1	0	0	1	1	3
Sahlstrand et al. 1978 [59]	1	1	0	0	1	1	4

*Item 1*: Is the control group representative for normal adolescents? 1 = Community control; 0 = Hospital controls/No description of source. *Item 2*: Was other pathology excluded that possibly influences the outcome? 1 = Yes; 0 = No or no description. *Item 3*: Were the same in- and exclusion criteria (except for the spinal deformity) used for AIS and healthy adolescents? 1 = Yes; 0 = No or no description. *Item 4*: Were the observers blinded to AIS/healthy adolescent status1 = Yes; 0 = No or not documented. *Item 5*: Was the data collection performed in the same standardised way for AIS cases and healthy adolescents? 1 = Yes; 0 = No or not documented. *Item 6*: Was the primary outcome parameter for AIS cases and healthy adolescents available? 1 = Available for > 90% of AIS and healthy adolescents, 0 = available for < 90% of AIS or healthy adolescents. Total score: Ranked according to total score; 0–2 high, 3–4 moderate and 5–6 low risk of bias

Table 5 outlines the results of the posturography methodological quality checklist. The appraisers identified 4 low (total score ≤ 49) [48, 52, 56, 58], 7 moderate (total score ≥ 50) [42–44, 46, 50, 54, 59] and 7 high-quality studies (total score > 80) [45, 47, 49, 51, 53, 55, 57].

**Table 5:** Overview of posturography methodological quality assessments of all included studies and total score

Author/year	Internal validity						Score	Statistical validity		Score	External validity					Score	Total score
	1	2	3a	3b	3c	3d		4	5		6a	6b	6c	6d	6e		
Sahli et al. 2013 [42]	+	+	+	–	+	+	83	+	–	50	+	–	+	+	+	80	71
Park et al. 2013 [43]	–	–	+	–	+	–	33	+	–	50	+	–	+	+	+	80	54
de Santiago et al. 2013 [44]	+	+	+	+	–	–	67	+	–	50	+	+	+	+	+	100	66
Stylianides et al. 2013 [45]	+	+	+	+	+	+	100	+	+	100	+	+	+	+	+	100	100
Gruber et al. 2011 [46]	+	–	+	+	–	+	66	+	–	50	+	+	+	+	+	100	72
Dalleau et al. 2011 [47]	+	+	+	+	+	+	100	+	+	100	+	+	+	+	+	100	100
Valles et al. 2009 [48]	+	+	–	–	–	–	33	+	–	50	–	–	–	–	–	0	28
Beaulieu et al. 2009 [49]	+	+	+	+	+	+	100	+	+	100	+	+	+	+	+	100	100
Zabjek et al. 2008 [50]	+	–	+	+	+	+	83	+	–	50	+	+	+	+	+	100	78
Dalleau et al. 2007 [51]	+	+	+	+	+	+	100	+	+	100	+	+	+	+	+	100	100
Simoneau et al. 2006 [52]	+	+	–	–	–	–	33	+	–	50	+	+	–	–	+	60	48
Chow et al. 2006 [53]	+	+	+	+	+	+	100	+	+	100	+	+	+	+	+	100	100
Simoneau et al. 2006 [54]	+	+	+	–	–	–	50	+	–	50	+	+	–	–	+	60	53
Allard et al. 2004 [55]	+	+	+	+	+	+	100	+	+	100	+	+	+	+	+	100	100
Silferi et al. 2004 [56]	+	+	–	–	–	–	33	+	–	50	+	+	–	–	–	40	41
Nault et al. 2002 [57]	+	+	+	+	+	+	100	+	+	100	+	+	+	+	+	100	100
Chen et al. 1998 [58]	–	+	–	–	–	–	17	–	–	50	+	+	–	–	+	60	42
Sahlstrand et al. 1978 [59]	+	+	–	+	–	–	50	+	+	100	+	+	–	–	–	40	63

Items indicate: *Internal validity* (1) reliability of outcome measures; (2) clear presentation of balance assessment; (3a) study controls for age; (3b) study controls for gender; (3c) study controls for height; (3d) study controls for body mass; *Statistical validity* (4) use of appropriate statistical tests; (5) adequate sample size; *External validity* (6a) Adequate information regarding age; (6b) adequate information regarding gender; (6c) adequate information regarding height; (6d) adequate information regarding body mass; (6e) adequate information regarding severity of curve (Cobb angle)

### Participants

The population, setting of recruitment, anthropometric measures, and scoliosis characteristics such as Cobb angle and primary curve type for AIS participants are summarised in Table 6. This review included a total of 954 participants (402 CON; 552 AIS) with mean age of 13.9 ± 1.8 for CON and 13.9 ± 1.9 for AIS. A total of 758 females (345 CON; 413 AIS) and 23 males (10 CON; 13 AIS) were included in the studies, and for the remaining 175 participants, gender was not reported [42, 43, 48]. A total of 13 studies reported the AIS mean Cobb angles, of which 4 studies [47, 49, 50, 53] participants were classified as mild with a distribution from 5° to 28°, 6 studies [43, 45, 46, 51, 55, 57] as moderate (25°–45°), 2 [52, 54] as severe (> 45°) and 1 as high severity (> 60°) [44]. Two studies reported Cobb angle ranges [42, 58], and for three studies, Cobb angles were not reported [48, 56, 59]. A total of 12 studies [42, 44–47, 49–53, 55, 57] reported a primary location or side of curvature, and right thoracic primary curves were predominant in 9 studies [44–47, 49, 51, 52, 55, 57].

**Table 6:** Participant characteristics of CON and AIS from all included studies

Author Year	Country and setting of participant recruitment	CON			AIS			
		*N* = Sample size, gender = ♀:♂	Age ± SD (years)	Height ± SD (cm), weight ± SD (kg)	*N* = Sample size, gender = ♀:♂	Cobb (°): range and/or mean ± SD, primary curve type	Age ± SD (years)	Height ± SD (cm), weight ± SD (kg)
Sahli et al. 2013 [42]	CON: Community, Tunisia AIS: Hospital, Tunisia	*N* = 12, gender = NR	13.2 ± 1.6	159.0 ± 8.0, 45.3 ± 6.6	*N* = 14, gender = 13:1	Range 10°–28°, *N* = 10 Right thoracic, *N* = 4 left lumbar	14.2 ± 1.6	160 ± 9.0, 51.8 ± 8.8
Park et al. 2013 [43]	CON: NR AIS: Hospital, South Korea	*N* = 15, gender = NR	14.7 ± 1.7	160 ± 6.0, 65.2 ± 10.4	*N* = 128, gender = NR	G1: *N* = 57, range 10°–20°, 13.7° ± 2.6 G2: *N* = 34, range 20°–30°, 23.9° ± 3.2 G3: *N* = 37, range > 40°, 36.4° ± 5.8	G1:15.5 ± 1.8 G2:15.1 ± 1.6 G3:15.5 ± 1.9	G1: 160 ± 6, 51.2 ± 9.1 G2: 160 ± 6, 47.3 ± 8.1 G3: 160 ± 7, 50.0 ± 8.5
de Santiago et al. 2013 [44]	CON: NR AIS: Hospital, Brazil	*N* = 15, gender = 15:0	15.1 ± 1.5	159.7 ± 0.0, 51.2 ± 2.0	*N* = 15, gender = 15:0	69.5° ± 8.78, right thoracic	15.0 ± 1.6	156.8 ± 0.03, 46.1 ± 3.3
Stylianides et al. 2013 [45]	CON: NR AIS: Hospital, Canada	*N* = 25, gender = 25:0	13.1 ± 1.4	156.9 ± 6.9, 46.0 ± 7.4	*N* = 28, Gender = 28:0	35.0° ± 7.6, right thoracic	12.9 ± 1.6	155.1 ± 9.9, 44.7 ± 9.5
Gruber et al. 2011 [46]	CON: NR AIS: Hospital, Mexico	*N* = 10, gender = 10:0	11.9 ± 2.8	149.0 ± 14.0, 44.5 ± 7.7	*N* = 36, gender = 36:0	G1: *N* = 18 pre-brace 27° ± 6, G2: *N* = 18 pre-op 52° ± 13, Right thoracic predominantly	12.5 ± 2.0	154 ± 11.0, 48.6 ± 12.5
Dalleau et al. 2011 [47]	CON: NR AIS: NR	*N* = 20, gender = 20:0	12.5 ± 1.3	156.3 ± 7.7, 43.7 ± 6.9	*N* = 21, gender = 21:0	range 5°–28°, 13.5° ± 5.5, Right thoracic	11.7 ± 3.1	148.4 ± 17.0, 40.0 ± 13.3
Valles et al. 2009 [48]	CON: NR AIS: NR	*N* = 20, gender = NR	NR	NR, NR	*N* = 16, gender = 13:3	NR	14.8 ± 2.1	151.9 ± 30.7, 59.8 ± 14.4
Beaulieu et al. 2009 [49]	CON: Community, Canada AIS: Hospital, Canada	*N* = 53, gender = 53:0	13.8 ± 1.0	159.2 ± 9.3, 49.9 ± 9.8	*N* = 49, gender = NR	G1: *N* = 23 OB, 18.9° ± 7.1, *N* = 20 Right thoracic, *N* = 3 Right lumbar G2: *N* = 26 PB, 27.2° ± 12.4, *N* = 25 Right thoracic, N = 1 Right lumbar	G1: 12.5 ± 2.4 G2: 12.2 ± 1.4	G1: 151.5 ± 10.7, 43.5 ± 10.9, G2: 152.3 ± 10.3, 42.1 ± 8.3
Zabjek et al. 2008 [50]	Canada, CON: NR, AIS: NR	*N* = 18, gender = 18:0	11.0 ± 2.0	144.0 ± 13.0, 39.0 ± 11.0	*N* = 22, gender = 22:0	21.0° ± 14.0, *N* = 8 Double, *N* = 7 Thoracolumbar, *N* = 7 Thoracic	12.2 ± 2.0	148.0 ± 11.0, 42.0 ± 12.0
Dalleau et al. 2007 [51]	CON: NR AIS: NR	*N* = 23, gender = 23:0	13.4 ± 1.0	161.5 ± 5.9, 50.0 ± 11.1	*N* = 23, gender = 23:0	29.4° ± 9.4, right thoracic	12.2 ± 1.5	154.0 ± 10.5, 44.4 ± 9.8
Simoneau et al. 2006 [52]	Canada, CON: NR, AIS: NR	*N* = 9, gender = 9:0	16.5 ± 1.7	NR, NR	*N* = 8, gender = 7:1	45.6° ± 7.5, right thoracic	16.3 ± 2.1	NR, NR
Chow et al. 2006 [53]	Canada, CON: NR, AIS: NR	*N* = 20, gender = 20:0	13.5 ± 1.1	155 ± 6, 44.8 ± 5.1	*N* = 26, gender = 26:0	21.0° ± 3.0, *N* = 10 right primary, *N* = 16 left primary	13.0 ± 0.9	156.0 ± 5.0, 43.9 ± 5.8
Simoneau et al. 2006 [54]	Canada, CON: NR, AIS: NR	*N* = 10, gender = 10:0	16.5	NR, NR	*N* = 8, gender = 7:1	45.6° ±7.5	16.4	NR
Allard et al. 2004 [55]	CON: Community AIS: NR	*N* = 36, gender = 36:0	13.5 ± 1.7	159.2 ± 8.4, 49.1 ± 10.3	*N* = 38, gender = 38:0	26.2° ± 11.5, right thoracic	12.4 ± 1.8	151.4 ± 11.2, 42.2 ± 9.3
Silferi et al. 2004 [56]	CON: NR AIS:NR	*N* = 15, gender = 15:0	range 11–16	NR, NR	*N* = 15, gender = 15:0	NR	range 11–16	NR, NR
Nault et al. 2002 [57]	Canada, CON: NR, AIS: NR	*N* = 38, gender = 38:0	12.9 ± 2.0	156.7 ± 10.8, 45.3 ± 8.5	*N* = 43, gender = 43:0	29° ± 12, range 7°–52°, *N* = 39 right thoracic, *N* = 2 thoracolumbar, *N* = 2 lumbar	12.5 ± 1.7	153.1 ± 9.7, 43.2 ± 9.1
Chen et al. 1998 [58]	CON: NR AIS: Hospital, Taiwan	*N* = 15, gender = 13:2	16.8 ± 3.1	NR, NR	*N* = 30, gender = 28:2	range 22°–67°	16.6 ± 3.8	NR, NR
Sahlstrand et al. 1978 [59]	CON: NR AIS: Hospital, Sweden	*N* = 48, gender = 40:8	13.4 ± 1.7	NR, NR	*N* = 32, gender = 27:5	NR	13.4 ± 2.1	NR

*G* group, *N* number, *NR* not reported, *OB* observation group, *PB* pre-bracing group, *SD* standard deviation

### Posturography method used to measure postural stability

The description of posturography method (Table 7) displays type of force plate, study protocol, COP parameters and values in each study. In 13 studies [45–57], the AMTI force plate (AMTI, Newton, MA, USA) was the most common to assess postural stability. A standard COP parameter sampling frequency ranged from 20 to 1080 Hz with 64 Hz cited in 8 studies [45, 47, 49, 51, 53, 55–57]. Two studies used 20 Hz [50] and 100 Hz [48] with two independent force plates under each foot. A variation in study protocols was observed with spacing of participant’s feet ranging from heels placed together [59] or shoulder width apart [44, 46]. Eleven studies specified the degree in which participant’s feet were externally rotated with 15° noted in 7 studies [45, 47, 49, 51, 55–57]. Disparity in trial durations and repetitions among the literature was evident. Trial duration varied from 10 s to 2 min, with 64 s being the most common [45, 49, 51, 55, 56]. The number of trials ranged from 1 to 6 trials. Three out of the 18 studies used study protocols which involved participants barefoot with heels spaced 23 cm apart, feet pointing externally 15°, vision focused on a target placed 1.2 m in front at eye level and three trials of 64 s [45, 51, 55].

**Table 7:** Description of data extracted from all included articles

Author Year	Force platform	Study protocol	COP parameters and direction of displacement	Values for AIS, CON, *p* value
Sahli et al. 2013 [42]	Static Stabilometric platform (SATEL, Blagnac, France), 40 Hz	Heels 5 cm apart, 30° angle between long axes of the feet. Eye focused at eye level, white cross on wall 2 m away, 3 trials of 51.2 s, 30-s rest interval	Mean ± SD Position of ML	#AIS = 22 ± 13.1 mm, #CON = 17 ± 26 mm, *p* < 0001*
			Mean ± SD Positon of AP	#AIS = 25 ± 37.4 mm, #CO*N* = 22 ± 22.5 mm, *p* < 0.001*
			Mean ± SD Sway Area	#AIS = 490 ± 318 mm^2^, #CON = 310 ± 364 mm^2^, *p* < 0.001*
Park et al. 2013 [43]	MFT balance tester (MFT balance test- basic, Multifunktionale trainsgerate, Germany)	Barefoot, subjects were to locate their centre of gravity into 5 different sections from the centre of a circle, 2 trials of 30 s	×Mean left and right balance in kg/m^2^ derived from the absolute values by subtracting the rate of the opposite direction from the rate of the selected direction	AIS G1 = 12.57 ± 9.55 kg/m^2^, G2 = 13.47 ± 11.54 kg/m^2^ G3 = 12.33 ± 10.68 kg/m^2^, CON = 2.38 ± 1.96 kg/m^2^, *p* < 0.01 between all groups*
			×Mean forward and backward balance in kg/m^2^ derived from the absolute values by subtracting the rate of the opposite direction from the rate of the selected direction	AIS G1 = 20.44 ± 12.91 kg/m^2^, G2 = 22.14 ± 18.03 kg/m^2^, G3 = 16.28 ± 11.43 kg/m^2^, CON = 10.37 ± 8.51 kg/m^2^, *p* < 0.01 between all groups*
de Santiago et al. 2013 [44]	EMG system force platform (Sao Jose dos Campos, Brazil), 100 Hz	Feet shoulder width apart, arms along body, eyes focused at eye level at 5 cm diameter black circle 1.5 m away, 3 trials of 60 s, self-chosen rest	xMean ± SD Sway Area	#AIS = 60 ± 1.4 mm^2^, #CO*N* = 40 ± 2.8 mm^2^, *p* < 0.0001*
Stylianides et al. 2013 [45]	AMTI force platform model OR6–5 (Newton, MA), 64 Hz	Barefoot, heels 23 cm apart, feet external rotated 15°, eyes focused at eye level target 1.2 m away, 3 trials of 64 s	Mean ± SD Range of ML	#AIS = 19.8 ± 9 mm, #CO*N* = 14.5 ± 6.3 mm, *p* < 0.05*
			Mean ± SD Range of AP, posterior displacement	#AIS = 32 ± 13.3 mm, #CON = 25.8 ± 7.8 mm, *p* < 0.05*
Gruber et al. 2011 [46]	AMTI force platform (AMTI, Newton, MA), 1080 Hz	Feet shoulder width apart, hands by the side, looking straight ahead, 3 trials of 10 s	Mean ± SD Range of ML	AIS = 28.99 ± 25.55 mm, CON = 17.25 ± 7.09 mm, *p* = 0.025*
			Mean ± SD Range of AP	AIS = 28.39 ± 11.44 mm, CON = 25.00 ± 11.72 mm, *p* = NS
			Mean ± SD Sway Area	AIS = 3.73 ± 0.40 mm^2^, CON = 3.48 ± 0.38 mm^2^, *p* < 0.005*
Dalleau et al. 2011 [47]	AMTI force platform model OR-5 (Newton, MA, USA), 64 Hz	Barefoot, heels 23 cm apart, feet pointing externally 15°, eyes focused on target 1.2 m ahead, 3 trials of 30 s	×Median (IQR) Range of ML	#AIS = 15.0 mm (6.5 mm), #CON = 12.0 mm (7.0 mm), *p* = 0.02*
			×Median (IQR) Range of AP, posterior displacement	#AIS = 25.5 mm (8.2 mm), #CON = 16.5 mm (7.2 mm), *p* < 0.01*
Valles et al. 2009 [48]	2xAMTI force plates under each foot model 0TS6–500, 100 Hz	3 trials of 30 s	×Mean ± SD Sway Area	AIS = 2728 ± 4177 mm^2^, CO*N* = 2152 ± 2767 mm^2^, *p* = NS
Beaulieu et al. 2009 [49]	AMTI force platform (Newton, MA, USA), 64 Hz	Heels spaced 20 cm apart, feet pointing externally 15°, eyes focused on target 1.2 m away, 3 trials of 64 s, 2-min rest intervals	Mean ± SD Position of ML, right displacement Mean ± SD Range of ML	#AIS = 4.58 ± 11.5 mm (OB & PB), #CON = 4.0 ± 9.0 mm, *p* = NS between OB, PB and CON groups. #AIS = 541.3 ± 244.9 mm, #CO*N* = 184.6 ± 153.8 mm, *p* < 0.001* OB vs CON, *p* < 0.001* PB vs CON
			Mean ± SD Position of AP, posterior displacement Mean ± SD Range of AP	#AIS = 73.8 ± 13.0 mm (OB & PB), #CON = 64.0 ± 13.0 mm, *p* = 0.04* OB vs CON, *p* = 0.001* PB vs CON #AIS = 629.0 ± 156.7 mm, #CO*N* = 233.3 ± 186.2 mm, p < 0.001* OB vs CON, *p* < 0.001* PB vs CON
			Mean ± SD Sway Area	#AIS = 245.0 ± 172.5 mm^2^ (OB & PB), #CON = 180 ± 115 mm^2^, *p* = NS OB vs CON, *p* = 0.008* PB vs CON
Zabjek et al. 2008 [50]	2× AMTI force platforms under each foot, 20 Hz	Quiet standing position, 4 trials of 2 min, adequate rest between each trial	×Mean ± SD Position estimate gravity line of COM ML, right displacement	AIS = 3.7 ± 11,08 mm, CON = 4.6 ± 9,69 mm, *p* = NR
			×Mean ± SD Position estimate gravity line of COM in AP, anterior displacement	AIS = 30.2 ± 16,06 mm, CON = 28.3 ± 16,16 mm, *p* = NR
Dalleau et al. 2007 [51]	AMTI force platform (Newton, MA, USA), 64 Hz	Barefoot, heels spaced 23 cm apart, feet pointing externally 15°, arms along body, eyes focused at eye level target 1.2 m away, 3 trials of 64 s, 60-s rest intervals	Mean ± SD Position of ML, right displacement Mean ± SD Range of ML	AIS = 3.3 ± 12.0 mm, CON = 3.4 ± 6.8 mm, *p* = NS #AIS = 18 ± 3.5 mm, #CON = 13 ± 2.5 mm, *p* = 0.001*
			Mean ± SD Position of AP, posterior displacement Mean ± SD Range of AP	AIS = 62.3 ± 10.7 mm, CON = 71.3 ± 14.3 mm, *p* = 0.043* #AIS = 29.5 ± 8 mm, #CON = 24 ± 7 mm, *p* = 0.016*
Simoneau et al. 2006a [52]	AMTI force platform model OR6–6 (Watertown, USA), 200 Hz	Barefoot with feet 10 cm apart, arms along body, eyes focused at eye level target 2 m away, 6 trials of 30 s divided into two 15-s intervals	×Mean distance between consecutive zones of 3 mm radius, sway density curve	#AIS = 3.8 mm ± 2,3, #CON = 1.69 mm ± 2,14, *p* < 0.01*
Chow et al. 2006 [53]	AMTI force platform (Newton, MA, USA), 64 Hz	Barefoot, heels 10 cm apart, feet pointing externally 30°, arms along body Eyes focused at eye level 10 cm × 15 cm reference square 2 m away, 3 trials of 60 s, 3-min rest intervals	×Mean Range of AP	AIS & CON = 31.9 mm averaged *p* = NR
Simoneau et al. 2006b [54]	AMTI force platform, 200 Hz	Barefoot with feet 10 cm apart, arms along body, eyes focused at eye level target 2 m away, 6 trials of 15 s	Mean ± SD Range of ML	#AIS = 13 ± 6.26 mm, #CON = 5.8 ± 6.39 mm, *p* = NR
			Mean ± SD Range of AP	#AIS = 19.5 ± 5.89 mm, #CON = 10.9 ± 6.1 mm, p = NR
Allard et al. 2004 [55]	AMTI force platform (Newton, MA, USA), 64 Hz	Heels spaced 23 cm apart, feet pointing externally 15°, eyes focused on target 1.2 m away, 3 trials of 64 s	Mean ± SD Position of ML, right displacement	AIS = 5.3 ± 14.2 mm, CON = 3.2 ± 9.3 mm, *p* = NS
			Mean ± SD Position of AP, posterior displacement	AIS = 26.1 ± 13.3 mm, CO*N* = 36.6 ± 12.4 mm, *p* = 0.002*
			Mean ± SD Sway Area	#AIS = 275 ± 175 mm^2^, CON = 183 ± 111 mm^2^, *p* = 0.010*
Silferi et al. 2004 [56]	AMTI force platform (Newton, MA), 64 Hz	Heels spaced 20 cm apart, feet external rotation 15°. Focused ahead at a target 1.2 m away, 3 trials of 64 s, 30-s rests	×RMS Amplitude, horizontal motion of ML	#AIS = 0.5 ± 0.5 mm, #CON = 0.2 ± 01 mm, *p* < 0.05*
			×RMS Amplitude, horizontal motion of AP	#AIS = 0.5 ± .2 mm, #CON = 0.7 ± 0.4 mm, *p* = NS
Nault et al. 2002 [57]	AMTI force platform (Newton, MA) at 64 Hz	Heels spaced 20 cm apart, feet external rotation 15°, arms along body, eyes focused at eye level target 1.2 m away, 3 trials of 60 s	Mean ± SD Position of ML, right displacement	AIS = 6.5 ± 10.1 mm, CON = 4.4 ± 9.1 mm, *p* = NS
			Mean ± SD Position of AP, posterior displacement	AIS = 72.7 ± 12.4 mm, CON = 85.0 ± 12.0 mm, *p* = 0.043*
			Mean ± SD Sway Area	AIS = 274.0 ± 154.0 mm^2^, CON = 190.3 ± 123.5 mm^2^, *p* = 0.009*
Chen et al. 1998 [58]	Kistler Instrument Corp at 50 Hz	Barefoot, feet parallel 8 cm apart 1 trial of 30 s	Mean ± SD Range ML	AIS = 17.0 ± 6.8 mm, CON = 13.4 ± 5.1 mm, *p* = 0.05*
			Mean ± SD Range AP	AIS = 25.9 ± 13.2 mm, CON = 20.4 ± 4.2 mm, *p* = NS
			Mean ± SD Sway Area	AIS = 765 ± 419 mm^2^, CON = 447 ± 98 mm^2^, *p* = 0.004*
Sahlstrand et al. 1978 [59]	Force platform L’Electronique Appliquee (Montrouge, France)	Heels together, feet external rotation 30°, eyes focused at eye level on a 10 × 10 cm reference square 5 m away, 1 trial of 2 min with 2-min rest intervals	×RMS ± SD Range of ML ×Mean ± SD Position of ML, right displacement	#AIS = 4.8 ± 1.38 mm, #CON = 4.4 ± 1.13 mm, *p* < 0.05* Data NR, *p* < 0.05*
			×RMS ± SD Range of AP ×Mean ± SD Position of AP	#AIS = 6.2 ± 1.9 mm, #CON = 5.6 ± 2.0 mm, *p* < 0.05* Data NR, *p* = NR
			×Mean ± SD Sway Area	#AIS = 107.1 ± 4.9 mm^2^, #CON = 78.5 ± 4.9 mm^2^, *p* < 0.05*

*AIS* adolescent idiopathic scoliosis, *AP* anteroposterior, *CON* typically developed adolescents, *COP* centre of pressure, *IQR* Inter quartile range, *ML* mediolateral, *NR* statistical significance not reported, *NS* no statistical significant, *OB* observation group, *PB* pre-bracing group; *p* value < 0.05 denotes statistical significance between groups*; *SD* standard deviation; vs = compared to; x = excluded from meta-analysis; # = results have been extracted from graph or figure

### Qualitative analysis of centre of pressure parameters

Description of data extracted from all included articles and findings of COP parameters and available statistical significance between groups are summarised in Table 7.

#### Sway area

Of 18 included studies, 9 investigated sway area [42, 44, 46, 48, 49, 55, 57–59] (Table 7). Seven studies reported significantly higher mean sway area values in AIS compared to CON [42, 44, 46, 55, 57–59]. One study [49] divided AIS subjects into pre-bracing (PB) and observation groups (OB). The PB group displayed 58% higher values than the CON, which was significant (*p* = 0.008). The OB showed 15% higher values than the CON; however, this was not a significant difference (NS).

#### Mediolateral

Of 18 included studies, 13 reported COP in ML position and range [42, 45–47, 49–51, 54–59] (Table 7). Two studies reported ML measurements as both position and range [49, 51]. Six studies reported position located towards the right [49–51, 55, 57, 59]; however, only one study [59] reported significant (*p* < 0.05) difference between AIS and CON. Eight studies reported higher values in the ML range for the AIS group compared to the CON with 7 studies reporting significant differences [45–47, 49, 51, 58, 59] and 6 noticed ML positional shift towards right [49–51, 55, 57, 59].

#### Anteroposterior

Of 18 included studies, 14 reported COP in AP position and range [42, 45–47, 49–51, 53–59] (Table 7). Two studies reported AP measurements both as position and range [49, 51]. Eight studies reported higher values in the AP range for the AIS group compared to the CON with 5 studies reporting significant differences [45, 47, 49, 51, 59]. The direction of AP position was significantly (*p* < 0.05) located posteriorly towards heels for AIS compared to CON in 5 of the included studies [42, 49, 51, 55, 57].

### Meta-analysis of COP parameters

To ensure consistency across the quantitative meta-analysis, a further 9 studies were removed due to divergent measurement units [43, 47, 52, 56], use of two separate force plates [48, 50], discrete values and graphs missing [53], due to high severity of scoliosis (mean 69.5° ± 8.78) [44] and procedure with heels together [59] (Fig. 1). Two of the excluded studies reported large ES differences in sway area in the qualitative analysis with evident postural instability for AIS compared to CON [44, 59]. Remaining 9 studies with a total of 491 participants (222 CON; 269 AIS) were included [42, 45, 46, 49, 51, 54, 55, 57, 58] with a summary of meta-analysis statistics presented in Table 8.

**Table 8:** Summary of meta-analysis statistics for selected COP parameters, 95% confidence interval (CI), heterogeneity assessed with Cochran’s Q and Higgin’s *I*^2^

COP parameters	Cohen’s d pooled effect size (95% CI)	Cochran’s Q	Higgin’s *I*^2^%	Studies (*n* = 9)
Sway area	0.65 (0.49–0.63) Medium	5.93	15.71	6
MLP1	0.15 (0.08–0.22) No difference	1.00	6.66	2
MLP2	0.14 (0.08–0.19) No difference	1.48	0.00	3
MLR	0.94 (0.83–1.04) Large	4.84	0.00	6
APP1	0.43 (0.31–0.54) Small	1.00	4.42	2
APP2	0.85 (0.72–0.97) Large	2.04	1.74	3
APR	0.98 (0.87–1.09) Large	6.35	21.30	6

*COP parameters*: Sway area, Mediolateral position 1 (MLP1), Mediolateral position 2 (MLP2), Mediolateral range (MLR), Anteroposterior position (APP1), Anteroposterior position 2 (APP2) and Anteroposterior range (APR). Cohen’s *d* pooled effect size difference defined as small *d* = 0.2, medium *d* = 0.5 and large *d* = 0.8

#### Sway area

**Fig. 2:**
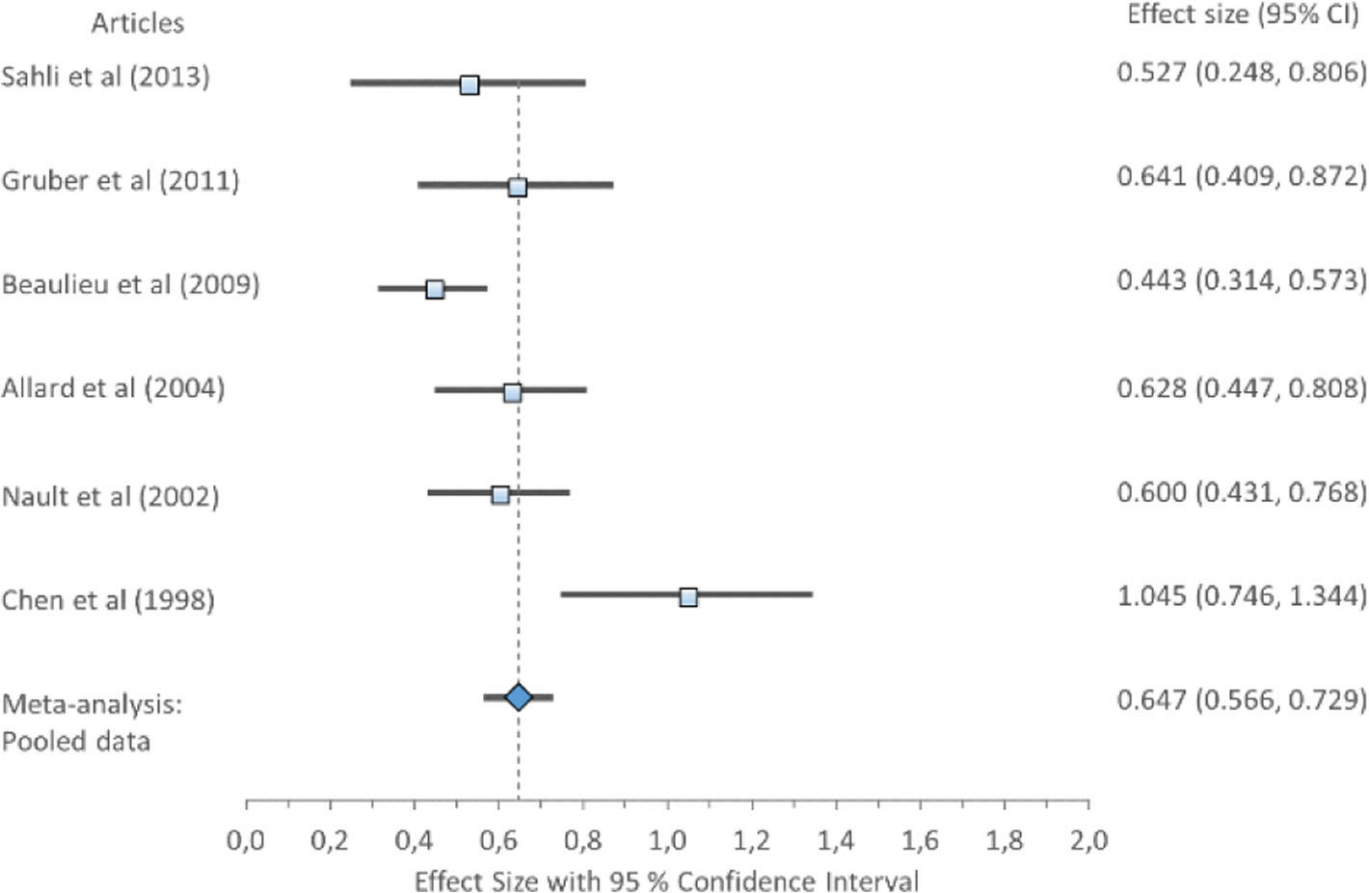
Forest plot of sway area with individual articles and Cohen’s *d* pooled data

The overall ES for sway area, Cohen’s *d* (pooled) showed a medium ES 0.65, 95% CI (0.49–0.63) (Table 8) displayed in Fig. 2. Forest plot of Sway area with Cohen’s *d* pooled effect size 0.65 (CI 0.49–0.63). Cochran’s Q 5.93 when compared to a chi-squares critical values table the *χ*^2^ of heterogeneity test was NS (*χ*^2^ = 11.071; df = 5; *p* = 0.05). Indicating that the variation between studies was homogeneous therefore accepting the null hypothesis that all studies are equal have a common ES. Further, the *I*^2^ statistic was 15.71% indicating low variability across studies, due to heterogeneity rather than chance. This suggests no observed heterogeneity across studies.

#### Mediolateral position and range

**Fig. 3:**
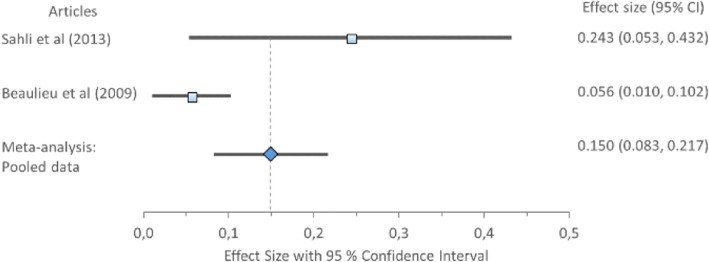
Forest plot of mediolateral position 1 (MLP1) with individual articles and Cohen’s *d* pooled data

**Fig. 4:**
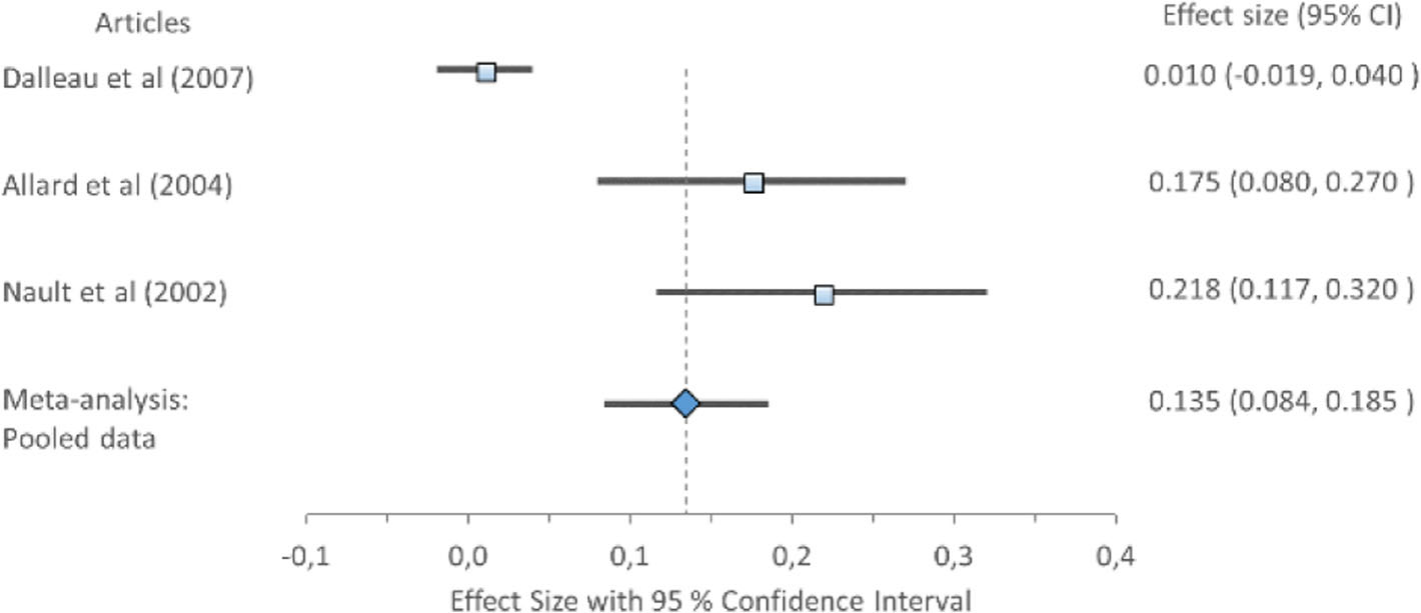
Forest plot of mediolateral position 2 (MLP2) with individual articles and Cohen’s *d* pooled data

**Fig. 5:**
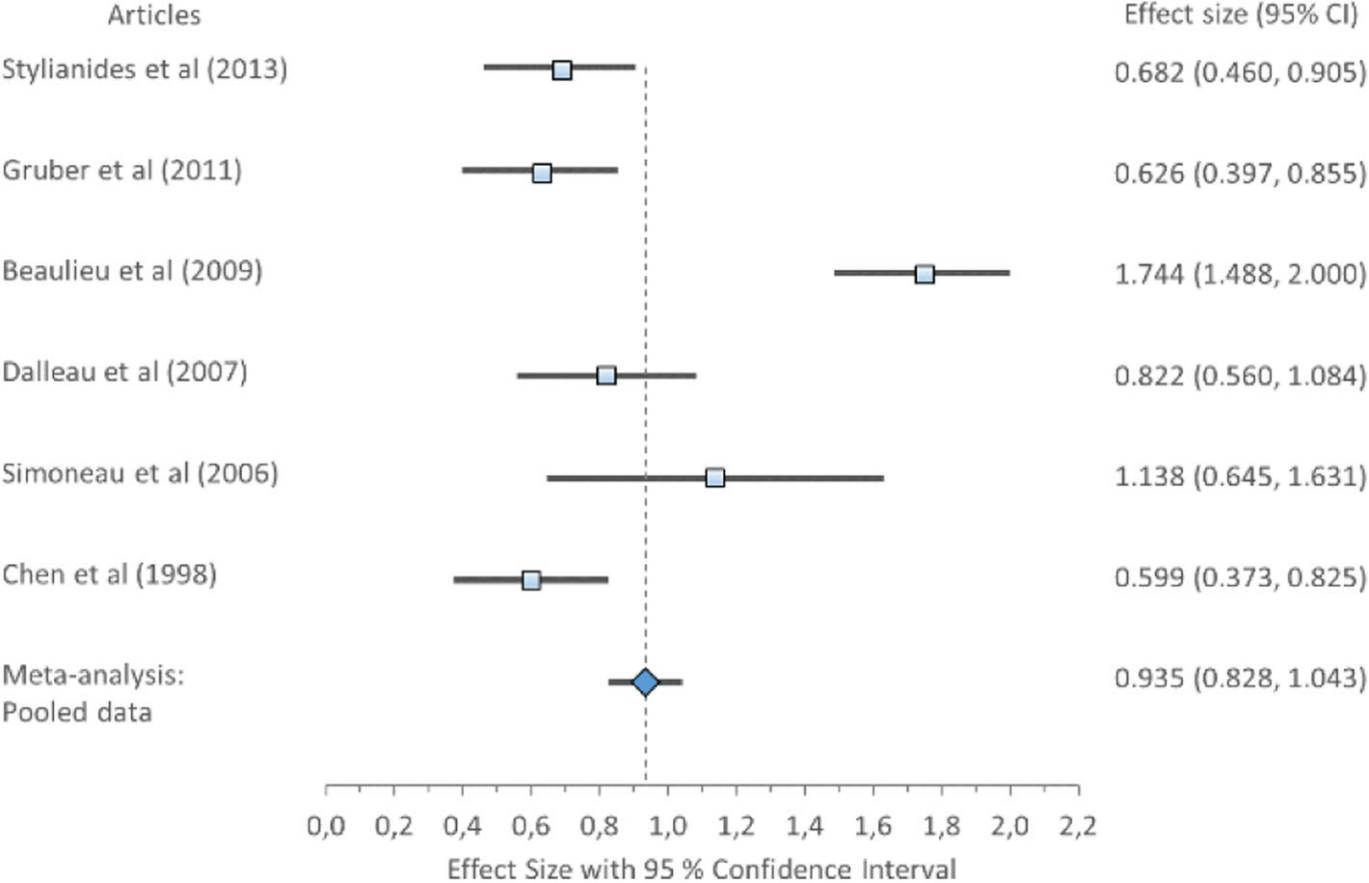
Forest plot of mediolateral range with individual articles and Cohen’s *d* pooled data

Cohen’s *d* (pooled) for MLP1 and MLP2 showed no ES difference but MLR showed large ES difference: MLP1 *0.15*, 95% CI (0.08–0.22); MLP2 *0.14*, 95% CI (0.08–0.19); and MLR 0.94, 95% CI (0.83–1.04) (Table 8) displayed in Fig. 3. Forest plot of Mediolateral position 1 (MLP1) with Cohen’s *d* pooled effect size 0.15 (CI 0.08–0.22), Fig. 4. Forest plot of mediolateral position 2 (MLP2) with Cohen’s *d* pooled effect size 0.14 (CI 0.08–0.19), Fig. 5. Forest plot of mediolateral range with Cohen’s *d* pooled effect size 0.94 (CI 0.83–1.04). Cochran’s Q showed that the variation between studies were homogeneous for each of the parameters MLP1 *1.00* (*χ*^2^ = 3.841; df = 1; *p* = 0.05), MLP2 *1.48* (*χ*^2^ = 5.991; df = 2; *p* = 0.05), and MLR *4.84* (*χ*^2^ = 11.071; df = 5; *p* = 0.05), consistent with Higgins *I*^2^ results of low variability, *6.66, 0*, and *0%*, respectively. This suggests no observed heterogeneity across studies, therefore accepting the null hypothesis that all studies are equal.

#### Anteroposterior position and range

**Fig. 6:**
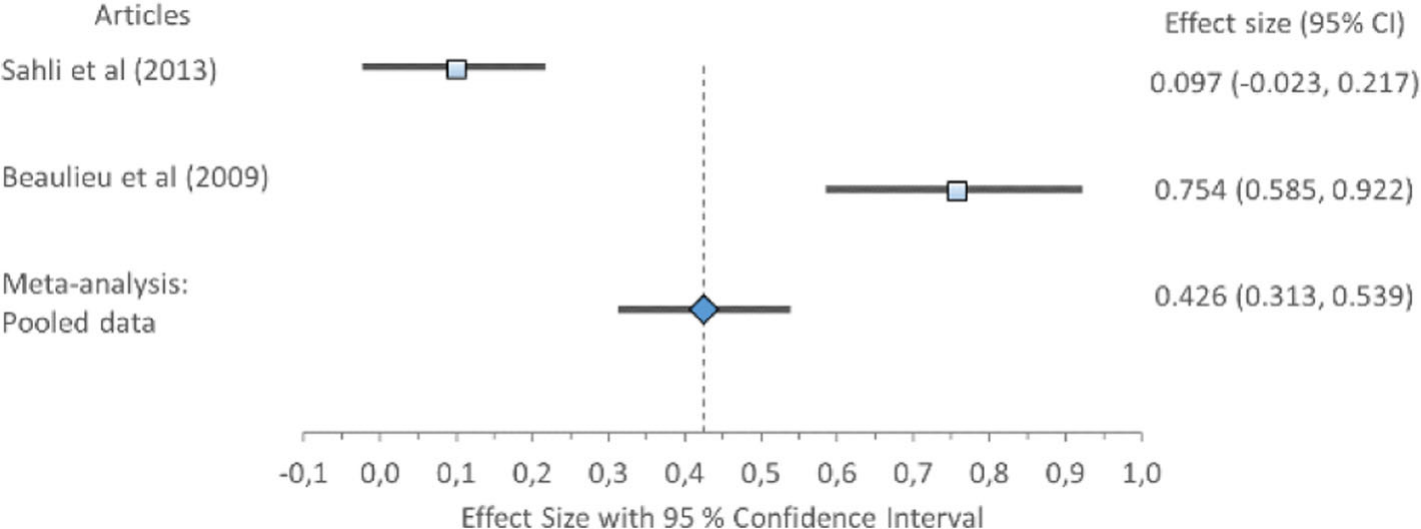
Forest plot of anteroposterior position 1 (APP1) with individual articles and Cohen’s *d* pooled data

**Fig. 7:**
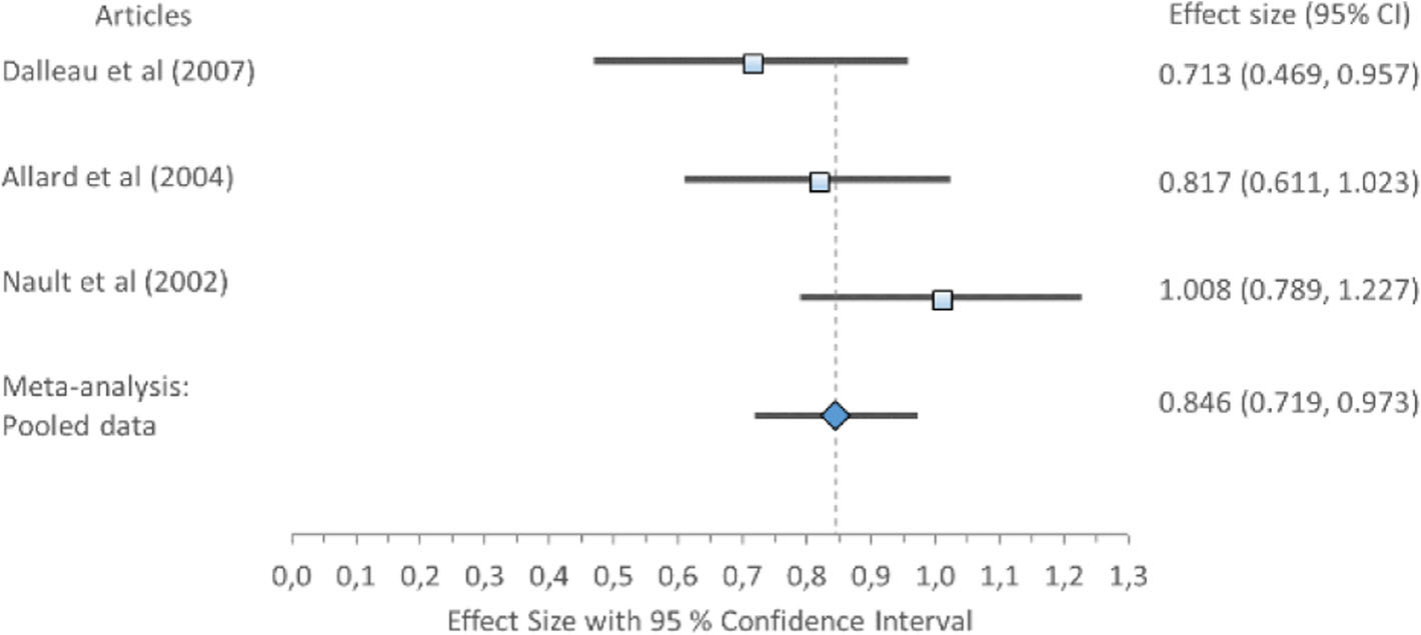
Forest plot of anteroposterior position 2 (APP2) with individual articles and Cohen’s *d* pooled data

**Fig. 8:**
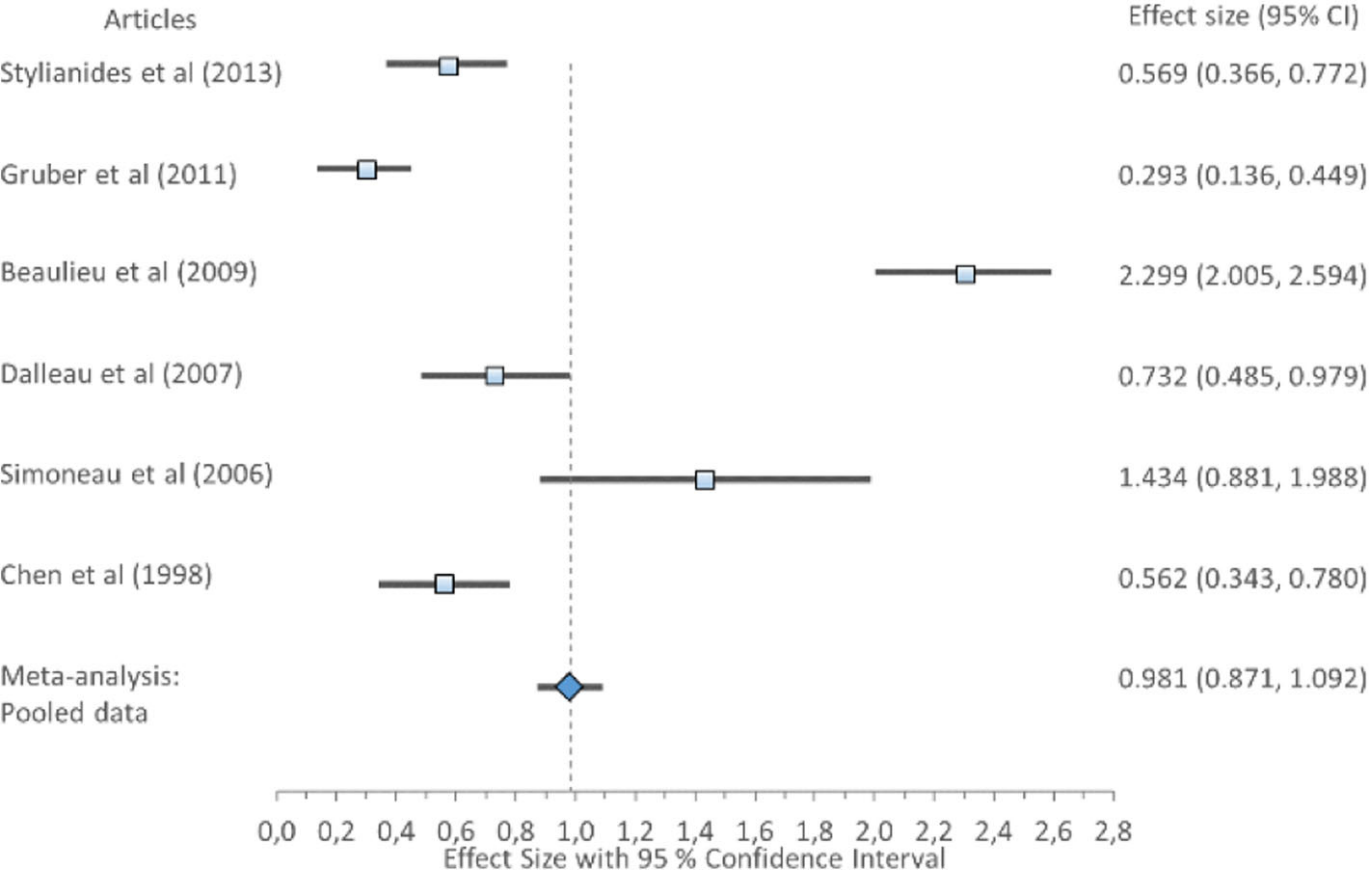
Forest plot of anteroposterior range (APR) with individual articles and Cohen’s *d* pooled data

Cohen’s *d* (pooled) for APP1 showed small ES difference but a large ES difference for APP2 and APR; APP1 *0.43*, 95% CI (0.31–0.54), APP2 *0.85*, 95% CI (0.72–0.97) and APR *0.98*, 95% CI (0.87–1.09) (Table 8) displayed in Fig. 6. Forest plot of anteroposterior position 1 (APP1) with Cohen’s *d* pooled effect size 0.43 (CI 0.31–0.54), Fig. 7. Forest plot of anteroposterior position 2 (APP2) with Cohen’s *d* pooled effect size 0.85 (CI 0.72–0.97), Fig. 8. Forest plot of anteroposterior range (APR) with Cohen’s *d* pooled effect size 0.98 (CI 0.87–1.09). Cochran’s Q showed that the variation between studies were homogeneous for each of the parameters; APP1 *1.00* (*χ*^2^ = 3.841; df = 1; *p* = 0.05), APP2 *2.04* (*χ*^2^ = 5.991; df = 2; *p* = 0.05), and APR *6.35* (*χ*^2^ = 11.071; df = 5; *p* = 0.05), consistent with Higgins *I*^2^ results of low variability across studies: APP1 *4.42%*, APP2 *1.74%* and APR *21.30%.* This suggests no observed heterogeneity across studies, therefore accepting the null hypothesis that all studies are equal.

## Discussion

This systematic literature review aimed to evaluate the current literature assessing COP parameters in AIS compared with CON to better understand and determine if AIS affects postural stability. According to the results from 7 different meta-analyses of COP parameters, AIS affects postural stability. In APP2, APR and MLR, a large ES difference was evident while sway area had medium ES difference for AIS compared to CON. This means larger postural instability (range and sway) for AIS with a COP positional shift posteriorly in the sagittal plane.

Although AIS is a three-dimensional spinal deformity, the lateral deviation of the spine in the coronal plane is often considered the most distinct deformity. Gruber et al. [46] reasoned that a reduced ML complexity reflected by a larger ML range indicates a more constrained, less adaptable postural control strategy in AIS. The qualitative synthesis showed a positional shift towards right in the frontal plane but also a posterior shift in the sagittal plane in some of the studies. However, when considering the levels of range and position for ML and AP COP parameters in the current review, ML and AP range ES difference was equivalently large. Furthermore, APP1 and APP2 position had greater ES difference compared to MLP1 and MLP2 having no ES difference. Therefore, a shift of COP position posteriorly in the sagittal plane may be considered as a more distinct effect of spinal curvature deformity. Nault et al. [57] investigated the hypothesis that COP parameters are related to standing stability parameters and found that a posterior inclination of the spine was commonly observed in scoliotic subjects. An AP position located closer to the back of the heels has been proposed in previous literature to be influenced by hypokyphotic posture [55]. Recently Leteneur [60] displayed that girls with right thoracic curvature classified as leaning backwards according to trunk sagittal inclination had greater COP range than those leaning forward which is consistent with the findings of present meta-analysis. Biomechanical studies have suggested that the human spine becomes more rotationally unstable with more dorsal shear loads in the thoracolumbar spine with backward trunk sagittal inclination postures [61]. This supports the importance of identifying AIS with deficits in postural stability and changed positional shift posteriorly in the sagittal plane and also testing the hypothesis that interventions focusing on scoliosis-specific postural correction as well as postural control may improve postural stability and potentially hinder spinal curvature progression.

An underlying aetiology of AIS is not well established within the current literature, as this condition is thought to be multifactorial in nature and no conclusive evidence exists to support any one theory [56]. Postural instability that presents at the onset of AIS has contributed to a dominant hypothesis that a deficiency in postural control results in scoliosis [15, 18, 62, 63]. Curve type, body segment orientation and body somatotype have all been identified as key factors that may perturb standing balance in the AIS population [55]. Poor postural stability exhibited by AIS patients has been described by two key hypotheses within the literature: a biomechanical and a sensory integration hypothesis. The biomechanical hypothesis gives importance to the biomechanical and morphological changes associated with AIS that are likely to lead to impaired postural stability [42, 64, 65]. These changes include the three-dimensional spinal curvature and deviations in the orientation of the head, shoulders, scapula and pelvis in all three planes [42].

The sensory integration hypothesis indicates individuals with AIS have impaired dynamic regulation of sensorimotor integration due to an inaccurate weighting of sensory inputs. This sensory deprivation has been linked with balance dysfunction reflected in an inability to recalibrate the position of the COP in relation to the body’s COM, and thus, exaggerated body sway oscillations are evident [15, 42]. A sensory integration disorder may also play an important role in curvature progression due to an inability to readjust COP position to counterbalance COM position over a long time scale [15]. Beaulieu et al. [49] suggest that greater neuromuscular demand is required in AIS to regulate body oscillations due to postural instability. A review studying associated abnormalities found a moderate level of evidence for impaired gait control in AIS [32]. The review summarised that the strength of evidence is low regarding different abnormalities in AIS showing that more research is needed to determine if a consistent pattern of abnormality exists [32]. What clinicians see in their examination of postural control is the net result of disease processes and the patients compensatory strategies in terms of behavioural components and adaptive plasticity in the nervous system [66]. We therefore need to differentiate between primary constraints on balance from compensatory strategies patients use to accomplish the goal of posture. Compensatory strategies may or may not be optimal or effective [67]. The findings of the current study support investigating postural stability and sensory integration in early stage AIS and prospectively to identify cause and effect of the curvature as well as effectiveness of postural control in the prevention of scoliosis progression.

The volume of studies included for analysis was a major strength of the present review. The total number of participants involved provided a greater power to the conclusions drawn from the literature. Furthermore, study participants were representative of the AIS female dominant gender distributions apparent in the community. Comparisons and conclusions are however limited by posturography methodological differences between studies and a need for standardisation of study protocols for future research. A review by Ruhe et al. [29] concluded that a minimum of 90-s sampling time, an average of three to five repetitions and sampling frequency of 100 Hz are required to reach acceptable reliability for most COP parameters. Therefore, we suggest that future studies assessing COP parameters in AIS adopt the above study protocol as baseline testing in unperturbed stance with the addition of participants’ feet spaced 23 cm apart, pointing externally 15°, and eyes focused on target 1.2 m ahead at eye level. More studies investigating sensory integration, with perturbed stance, are needed to allow for meta-analysis of data from studies testing proposed sensory integration hypothesis.

It is also important to consider potential limitations of the current review. For example, 2 of the studies in the meta-analyses showed a high risk of bias [54, 57], 5 moderate [45, 46, 51, 55, 58] and 2 low risk-of-bias [42, 49]. Furthermore, posturography methodological quality checklist displayed 1 study showing low [58], 3 moderate [42, 46, 54] and 5 high-quality scores for studies included in the meta-analyses [45, 49, 51, 55, 57]. Discrepant methodological quality may be explained by differing aims, sampling methods, AIS curve types and size heterogeneity as well as reporting of maturation characteristics. For example, some studies primarily aimed to investigate the effect of back pack load on COP parameters, differences in gait patterns and risk of falls within subgroups of AIS [42, 53, 58]. None of the studies discussed statistical power and there was a lack of consistency in reporting sampling as well as AIS classification. These factors may influence the variation around the COP parameter means within AIS samples, but one must also consider the inherent variation that exists within CON. Future research in this field would benefit from standardised reporting of sampling, posturography methods as well as anthropometrical and maturation characteristics of AIS and typically developed adolescent populations.

## Conclusion

There is moderate quality evidence for decreased postural stability in AIS measured as COP parameters sway area, ML and AP range with a positional shift posteriorly in the sagittal plane. The findings support studying postural stability in early stage AIS and also prospectively identify cause and effect of the curvature as well as effectiveness of postural control interventions in the prevention of scoliosis progression.

## Abbreviations

**♀**: Female
♂: Male
AIS: Adolescent Idiopathic scoliosis
AMTI: Advanced Mechanical Technology Incorporated
AP: Anteroposterior
APP: Anteroposterior position
APR: Anteroposterior Range
BOS: Base of support
CI: Confidence interval
CINAHL: The Cumulative Index of Nursing and Allied Health Literature database
COG: Centre of gravity
COM: Centre of body mass
CON: Typically developed adolescents as control group
COP: Centre of pressure
DF: Degrees of freedom
ES: Effect size
GRF: Ground reaction forces
Hz: Herz, unit of frequency defined as 1 cycle per second
ML: Mediolateral
MLP: Mediolateral position
MLR: Mediolateral range
NR: Not reported
NS: No statistical significance
OB: Observation groups
PB: Pre-bracing
PEDro: Physiotherapy Evidence Database
PRISMA: The Preferred Reporting Items for Systematic Reviews and Meta-Analyses guidelines
PubMed: Publishers Medline by the National Library of Medicine journal citation database
S: Statistical significance
Scopus: Abstract and indexing database by Elsevier
SD: Standard deviation
SE: Standard error
SEM: Standard error of measurements
